# Oral Antibiotic Prophylaxis Reduces Surgical Site Infection and Anastomotic Leakage in Patients Undergoing Colorectal Cancer Surgery

**DOI:** 10.3390/biomedicines9091184

**Published:** 2021-09-09

**Authors:** Simran Grewal, J. Reinder D. Reuvers, Gabor S. A. Abis, René H. J. Otten, Geert Kazemier, Hein B. A. C. Stockmann, Marjolein van Egmond, Steven J. Oosterling

**Affiliations:** 1 Department of Molecular Cell Biology and Immunology, Amsterdam University Medical Centers, De Boelelaan 1108, 1081 HZ Amsterdam, The Netherlands; j.reuvers@amsterdamumc.nl (J.R.D.R.); m.vanegmond@vumc.nl (M.v.E.); 2 Department of Surgery, Cancer Center Amsterdam, Amsterdam University Medical Centers, De Boelelaan 1117, 1081 HV Amsterdam, The Netherlands; g.kazemier@amsterdamumc.nl; 3 Department of Surgery, Spaarne Gasthuis, Boerhaavelaan 22, 2035 RC Haarlem, The Netherlands; gaborabis@gmail.com (G.S.A.A.); stockmann@spaarnegasthuis.nl (H.B.A.C.S.); sj.oosterling@spaarnegasthuis.nl (S.J.O.); 4 Medical Library, Amsterdam University Medical Centers, De Boelelaan 1117, 1081 HV Amsterdam, The Netherlands; reneotten1954@gmail.com

**Keywords:** colorectal carcinoma, surgery, oral antibiotics, surgical site infection, anastomotic leakage

## Abstract

Background: Surgical-site infection (SSI) and anastomotic leakage (AL) are major complications following surgical resection of colorectal carcinoma (CRC). The beneficial effect of prophylactic oral antibiotics (OABs) on AL in particular is inconsistent. We investigated the impact of OABs on AL rates and on SSI. Methods: A systematic review and meta-analysis of recent RCTs and cohort studies was performed including patients undergoing elective CRC surgery, receiving OABs with or without mechanical bowel preparation (MBP). Primary outcomes were rates of SSI and AL. Secondarily, rates of SSI and AL were compared in broad-spectrum OABs and selective OABs (selective decontamination of the digestive tract (SDD)) subgroups. Results: Eight studies (seven RCTs and one cohort study) with a total of 2497 patients were included. Oral antibiotics combined with MBP was associated with a significant reduction in SSI (RR = 0.46, 95% confidence interval (CI) 0.31–0.69), I^2^ = 1.03%) and AL rates (RR = 0.58, 95% CI 0.37–0.91, I^2^ = 0.00%), compared to MBP alone. A subgroup analysis demonstrated that SDD resulted in a significant reduction in AL rates compared to broad-spectrum OABs (RR = 0.52, 95% CI 0.30 to 0.91), I^2^ = 0.00%). Conclusion: OABs in addition to MBP reduces SSI and AL rates in patients undergoing elective CRC surgery and, more specifically, SDD appears to be more effective compared to broad-spectrum OABs in reducing AL.

## 1. Introduction

Colorectal carcinoma (CRC) surgery is still associated with significant infectious morbidity despite advances in recent decades in surgical techniques and antiseptic measures. With 1.8 million people diagnosed with CRC in 2018, this clinical problem remains a serious issue in surgical healthcare [1]. Surgical site infection (SSI) and anastomotic leakage (AL) affect around 20% of patients [2]. AL is the most severe complication, with an incidence ranging from 2 to 19% [3] and a mortality rate of 1.7–16.4% [4,5]. 

The vast majority of SSIs are caused by endogenous bacteria residing in the digestive tract, of which potentially pathogenic microorganisms (PPMs) account for most hospital-acquired infections [6,7]. These microorganisms are part of the gut microbiota’s composition, which is highly variable between different individuals. As with the microbiome itself, the variability also accounts for the number of PPMs [8,9]. This group of microorganisms consists mostly of predominant aerobic Gram-negative bacteria such as *E. coli, Klebsiella, Proteus, and Enterobacter species* [10,11]. In healthy individuals, the dense intestinal mucus layer prevents PPMs from gaining access to host tissues to initiate inflammation. As the gastrointestinal tract is opened and the intestinal epithelium disrupted during colorectal surgery, the surgical site can easily be contaminated with these PPMs. In addition, the composition of the intestinal flora changes following colorectal surgery, with increased counts of PPMs [12], having possible negative effects for the host [13,14]. This suggests that the individual composition of the microbiome and peri-operative variations of its configuration, explained by various causes such as mechanical bowel preparation (MBP), antibiotics, and colorectal surgery itself, may have important effects on the patient’s course [15,16,17,18]. Therefore, peri-operative measures that modulate the intestinal microbial composition may be of clinical relevance. Intravenous (i.v.) antibiotic prophylaxis is considered the standard of care, with strong evidence for its benefits in terms of reducing surgical site infection and overall mortality [19]. In addition, long-standing areas of controversy between surgeons globally are the use of MBP and/or prophylactic oral antibiotics (OABs) prior to elective colorectal resection. Several clinical studies and meta-analyses have been published on MBP and OABs, in addition to i.v. antibiotics prophylaxis before colorectal surgery. Unfortunately, the prescribed MBP and/or antibiotic regimes vary, making comparison between these studies complex [19,20,21,22,23,24,25,26,27]. Moreover, the clinical benefit of the protective role of OABs against infectious complications is still interpreted in the context of combined preparations [25,27,28] and little is known regarding the potential benefits of OABs alone [28,29,30]. Furthermore, in most of the studies evaluating the effect of MBP and/or OABs, including a recently published meta-analysis [31], the reason for surgery varied considerably, from diverticulitis and fistula to oncological resections. To address these limitations, we conducted a meta-analysis of recent studies on the impact of OABs on rates of SSI and AL, in patients undergoing elective surgical resection of colorectal cancer. 

Our aim was to investigate the role of OAB with or without MBP on the rates of these infectious complications. Furthermore, a subgroup analysis was performed to evaluate the effect of selective OABs and broad-spectrum OABs. 

## 2. Materials and Methods

### 2.1. Search Strategy 

A literature search was performed by R.O, based on the Preferred Reporting Items for Systematic Reviews and Meta-Analysis (PRISMA)-statement (www.prisma-statement.org; accessed on 1 March 2020) [32]. To identify all relevant publications about the use of antibiotics for the bowel preparation in colorectal surgery, systematic searches were performed in the bibliographic databases PubMed (https://pubmed.ncbi.nlm.nih.gov), EMBASE.com (https://www.embase.com), The Cochrane Library (https://www.cochranelibrary.com), Web of Science (https://www.webofknowledge.com, and Google Scholar (https://scholar.google.com) from the past 20 years (1 January 2000 till 1 March 2020).

Search terms included controlled terms (MeSH in PubMed and Emtree in Embase) and free text terms. We used free text terms only in The Cochrane Library. In Embase we excluded conference abstracts. Search terms expressing “colon/colorectal surgery” were used in combination with search terms comprising “antibiotics” and search terms comprising “bowel preparation”. The references of the identified articles were searched for relevant publications. 

### 2.2. Selection of Articles

Articles were screened for suitability on the basis of the title and abstract by 2 independent researchers (R.R and S.G). Studies were eligible for inclusion if they examined the role of OABs preparation with or without MBP added to standard i.v. prophylaxis and compared these to either MBP alone, OABs alone, or no other preparation added to the standard i.v. prophylaxis in adult patients due to undergoing elective colorectal surgery for colorectal cancer (a minimum of 95% of the patient population in the study). At least one relevant clinical outcome was reported. Only RCTs and cohort studies were included. The bibliographies of all studies which met the inclusion criteria, and previous systematic reviews and meta-analyses on the subject, were reviewed to ensure study inclusion was as complete as possible. Exclusion criteria were: studies which did not consider any relevant clinical outcomes, non-English language publications, and studies performed in animals or children.

### 2.3. Endpoints

The primary endpoints were the rates of SSI and AL. A subgroup analysis was performed to determine whether incidences of these infectious complications differed in selective OABs compared to broad-spectrum OABs.

### 2.4. Antibiotic Definitions

OABs can be divided into: (a) broad-spectrum OABs, which can inhibit a wide variety of aerobe and anaerobe bacteria; and (b) selective OABs, which are antibiotics that are primarily effective against a specific group of microbes, i.e., aerobe Gram-negative bacteria. The selective decontamination of the digestive tract (SDD) is also mentioned in some of the included studies. This is an orally administered combination of non-absorbable antibiotics to decontaminate the digestive tract of aerobic Gram-negative rods and yeasts, while preserving anaerobic microbiota [17].

### 2.5. Data Extraction and Management

The two reviewers (R.R. and S.G) independently extracted data from each outcome for the published results of included trials. Any discrepancies were resolved by the senior authors (M.v.E., S.O., and H.S). Baseline characteristics of the study, and the number of patients randomized and analyzed, were retrieved. Data were also collected on the type of resection, open versus laparoscopic surgery, and details of the preparation used, in terms of i.v. antibiotic and OAB, and MBP. When data on a specific endpoint were not provided in the article, an attempt was made to contact the authors to clarify details and/or to request missing data on outcome. The risk of bias was assessed for the included RCTs using the Cochrane Collaboration Tool (Cochrane.org), which considers random sequence generation (selection bias), allocation concealment (selection bias), blinding of participants and clinicians/researches (performance bias), blinding of outcome assessment (detection bias), incomplete outcome data (attrition bias), and selective reporting (reporting bias)[33]. 

### 2.6. Statistical Analysis 

For the included studies, pooled risk ratios (RRs) were calculated with 95% confidence intervals (c.i) using a random effects model, after the raw risk ratio was log transformed and finally back transformed to the original scale. Heterogeneity of trial results was tested with the I^2^ statistic to provide an estimate of the degree of heterogeneity. I^2^ values over 50% indicate considerable heterogeneity [34]. Outcomes were analyzed using R software (2019): a language and environment for statistical computing (R Foundation for Statistical Computing, Vienna, Austria. Version 3.5.3).

## 3. Results

**Figure 1:**
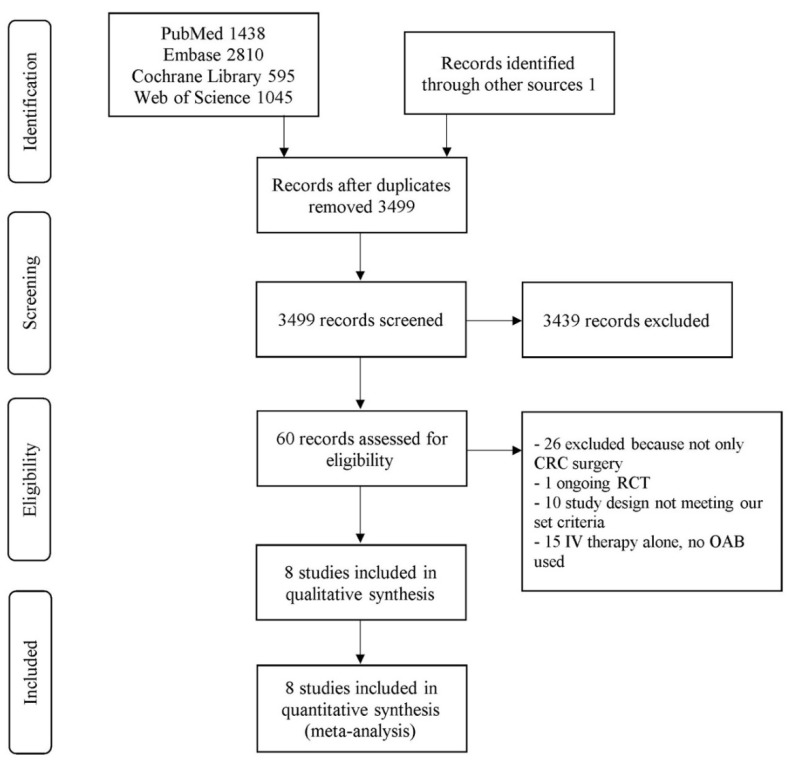
PRISMA diagram showing identification of studies from the initial literature search.

Titles and abstracts of 3499 studies were retrieved from PubMed, Embase, Cochrane Library database, Web of Science, and Google Scholar searches. Primary inclusion criteria were met in 60 studies, which were subsequently reviewed in detail. A total of 52 articles failed to meet the inclusion criteria and were therefore excluded: 26 studies did not include patients exclusively undergoing elective CRC surgery, one study was an ongoing RCT, 10 studies were neither a RCT nor a cohort study, and 15 studies only used i.v. therapy. Eight publications were eligible for inclusion with a total of 2497 patients (Figure 1). Of these, seven studies were RCTs with 2335 participants, and one was a cohort study with 162 patients [17,35,36,37,38,39,40,41]. The baseline characteristics per study, including patient demographics and surgical variables, and the details of antibiotic regimes administered, are presented in Table 1.

### 3.1. Quality Assessment and Patient Demographics

The risk of bias in the RCTs included was variable, from well-documented random sequence generation and allocation concealment to a high risk of bias concerning blinding procedures (Table 2).

#### 3.1.1. Antibiotic Regimes

Of the 2497 patients, 1240 received preoperative OABs with MBP in combination with i.v. antibiotic prophylaxis (intervention group), and 1257 patients received MBP and standard i.v. antibiotics (control group). All trials used systemic antibiotics with Gram-negative coverage, with the exception of the antibiotics administered to the control group in the study of Schardey et al. [41]. Seven studies used a cephalosporin (first or second generation), in two studies combined with metronidazole. Schardey et al., did not use a cephalosporin for their systemic treatment, but instead administered amphotericin B and lactulose.

The oral prophylactic regimen in the intervention groups differed between trials. Broad-spectrum OABs, consisting of kanamycin with either metronidazole or erythromycin, targeting both Gram-positive and Gram-negative bacteria, were given before surgery in five trials [35,36,37,38,39]. Selective decontamination of the digestive tract (SDD) using the combination of polymyxin, tobramycin, and amphotericin was given in the remaining three trials [17,33,41]. In the study of Schardey et al., vancomycin was added to the oral regime [41]. Data are summarized in Table 1.

#### 3.1.2. MBP

All studies, with the exception of one, investigated the role of OABs in the context of MBP. Therefore, the specific impact of MBP could not be determined. In the study conducted by Abis et al., MBP was given for left-sided colonic, sigmoid, and low anterior resections only; however, no prespecified subgroup analyses were planned for right versus left colectomies in other studies. The type of bowel preparation varied between studies and consisted of sodium picosulphate and magnesium citrate [38,39], polyethylene glycol [35,36,37], or Klean Prep [17,39,41] (Table 1).

#### 3.1.3. Type of Surgery

Three studies [33,39,40] focused on surgery using laparoscopic techniques, two trials [35,40] on open surgery alone, and three studies did not provide this information [17,37,38].

### 3.2. Primary Outcome: Anastomotic Leakage and Surgical Site Infection

#### 3.2.1. Anastomotic Leakage (AL)

**Figure 2:**
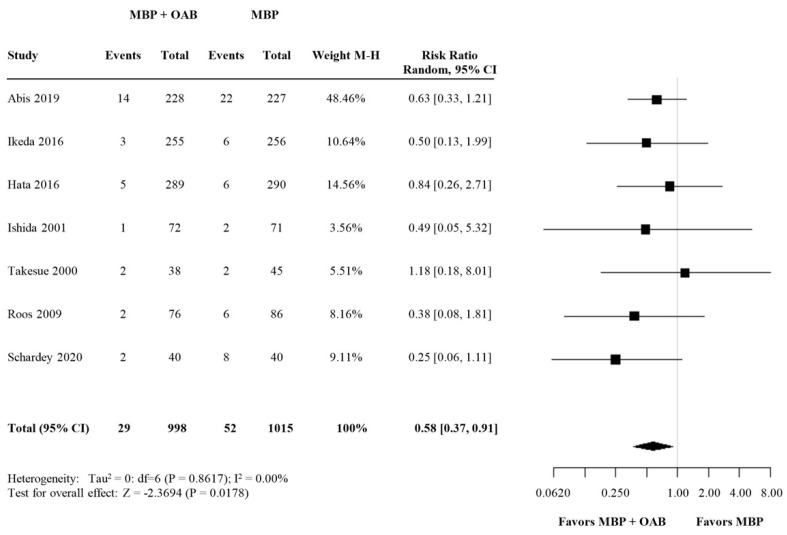
Forest plot comparing anastomotic leakage rate for patients receiving OABs in addition to MBP and i.v. antibiotic prophylaxis versus MBP alone with i.v. antibiotic prophylaxis. A random effects model was used to perform the meta-analysis and risk ratios are quoted including 95% confidence intervals.

In total, eight studies compared the rates of AL in patients who were given preoperative OAB + MBP + i.v. antibiotic prophylaxis (intervention group), with those who received only MBP + i.v. antibiotic prophylaxis (control group). Data are summarized in Figure 2. 

AL occurred in 29 of 998 patients in the intervention group (2.9%) versus 52 of 1015 patients in the control group (5.1%). There was no heterogeneity between the included studies (I^2^ = 0.00%). Meta-analysis of the included studies showed a statistically significant reduction of AL in the group of patients receiving OABs in addition to MBP + i.v. antibiotics (RR = 0.58, 95% CI 0.37–0.91) (Figure 2). None of these trials compared OAB + MBP + i.v. antibiotics versus OAB alone, or OAB + MBP versus i.v. antibiotics alone.

#### 3.2.2. Surgical Site Infection (SSI)

In the five RCTs and one cohort study, SSI rates in patients given preoperative OABs in addition to MBP and standard i.v. antibiotic prophylaxis, were compared with those in patients who received only MBP + i.v. antibiotic prophylaxis. None of the trials compared OAB + MBP combined + i.v. antibiotic prophylaxis versus OAB alone or OAB + MBP versus i.v. antibiotics alone.

**Figure 3:**
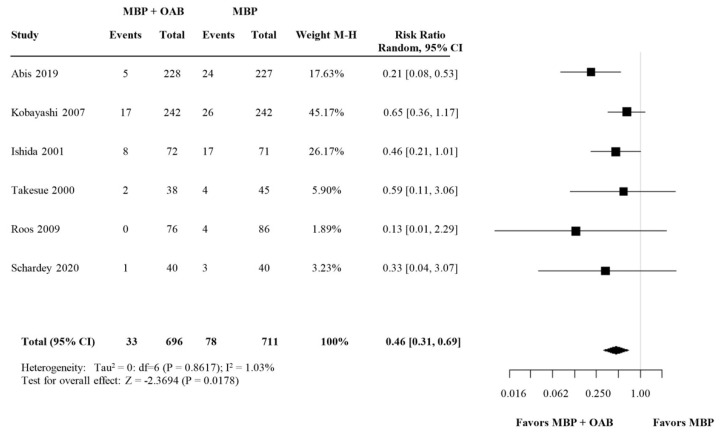
Forest plot comparing SSI rate for patients receiving OABs in addition to MBP and i.v. antibiotic prophylaxis versus MBP with i.v. antibiotic prophylaxis. A random effects model was used to perform the meta-analysis and risk ratios are quoted including 95% confidence intervals.

Infectious complications occurred in 33 of 696 patients in the OAB + MBP + i.v. antibiotic group (4.7%) versus 78 of 711 patients in the MBP + i.v. antibiotic group (11%). There was very low heterogeneity between the included studies (I^2^ = 1.03%). Meta-analysis of the included studies showed a statistically significant protective effect of OAB in addition to MBP + i.v. antibiotic prophylaxis on SSI versus MBP + i.v. antibiotic prophylaxis alone (RR = 0.46, 95% CI (0.31–0.69)) (Figure 3).

#### 3.2.3. Subgroup Analysis: SDD versus Broad-Spectrum OABs

**Figure 4:**
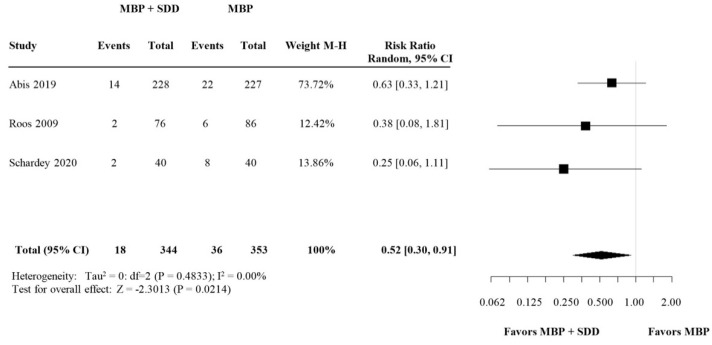
Forest plot comparing anastomotic leakage rate for patients receiving SDD (selective decontamination of the digestive tract is selective OAB targeting only the specific aerobe, Gram-negative bacteria) in addition to MBP and i.v. antibiotic prophylaxis versus MBP with i.v. antibiotic prophylaxis. A random effects model was used to perform the meta-analysis and risk ratios are quoted including 95% confidence intervals.

**Figure 5:**
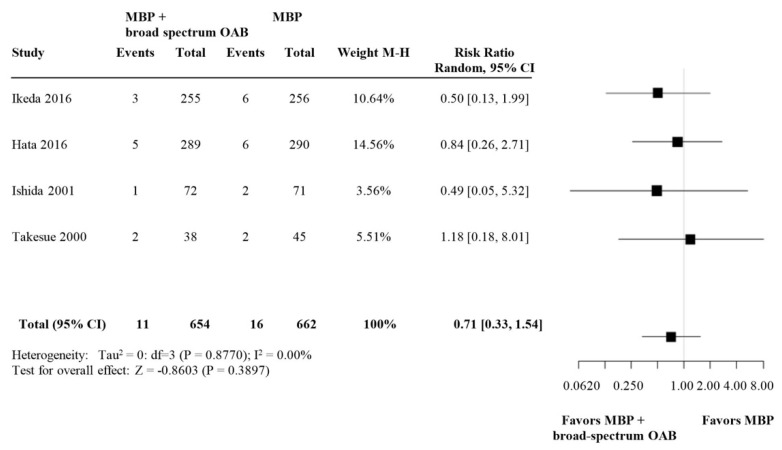
Forest plot comparing anastomotic leakage rate for patients receiving broad-spectrum OABs (defined as OABs that are effective against both aerobe and anaerobe bacteria) in addition to MBP and i.v. antibiotic prophylaxis versus MBP alone with i.v. antibiotic prophylaxis. A random effects model was used to perform the meta-analysis and risk ratios are quoted including 95% confidence intervals.

A subgroup analysis of selective decontamination of the digestive tract (SDD) versus broad-spectrum antibiotics showed that SDD was associated with a significant reduction in AL rates, whereas this effect was not significant in broad-spectrum antibiotics (RR 0.52, (95% CI 0.30–0.91) versus RR 0.71, (95% CI 0.33–1.54)) (Figure 4 and Figure 5, respectively).

**Figure 6:**
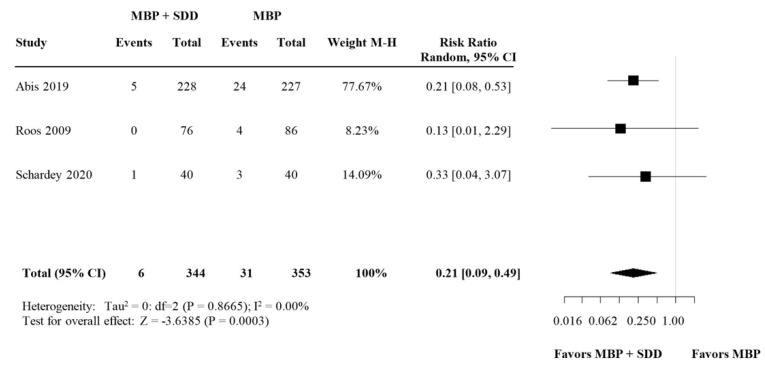
Forest plot comparing SSI rate for patients receiving SDD (selective decontamination of the digestive tract is selective OAB targeting only specific the aerobe, Gram-negative bacteria) in addition to MBP and i.v. antibiotic prophylaxis versus MBP with i.v. antibiotic prophylaxis. A random effects model was used to perform the meta-analysis and risk ratios are quoted including 95% confidence intervals.

**Figure 7:**
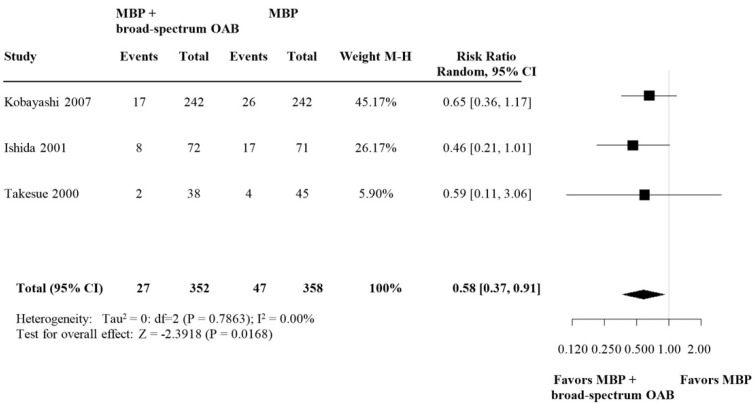
Forest plot comparing SSI rate for patients receiving broad-spectrum OABs (defined as OAB that are effective against both aerobe and anaerobe bacteria) in addition to MBP and i.v. antibiotic prophylaxis versus MBP alone with i.v. antibiotic prophylaxis. A random effects model was used to perform the meta-analysis and risk ratios are quoted including 95% confidence intervals.

In the subgroup analysis, a significant reduction of SSI in both variants of OAB regimes was seen; SDD (RR 0.21, 95% CI (0.09–0.49) compared to broad-spectrum antibiotics (RR 0.58, 95% CI (0.37–0.91) (Figure 6 and Figure 7 respectively).

## 4. Discussion

The present systematic review and meta-analysis demonstrate that preoperative SDD prophylaxis in combination with MBP and i.v. antibiotic prophylaxis is associated with a significant reduction in rates of both SSI and AL in patients undergoing elective resection for colorectal cancer. To our knowledge, this study is the first meta-analysis in this selected group of patients showing a significant effect of OABs on AL.

Reduced rates of SSI and, to some extent, AL in patients receiving OABs plus MBP were shown in earlier studies [17,22,27,31,38,42,43,44,45,46,47,48,49,50]; however, indications for surgery varied considerably in these studies and did not only include CRC, but also inflammatory bowel disease (IBD), fistulas, and other benign and/or infectious diseases that have a different post-operative complication risk. Furthermore, patients treated for IBD often used immunosuppressive medication, which influenced the incidence on infectious complications and tissue healing. The present meta-analysis comprised a more homogenous patient group, focusing only on patients undergoing elective resection for colorectal cancer. Furthermore, to ensure the quality of our results, it was decided to include studies published after 2000, because implementation of enhanced recovery after surgery (ERAS) protocols and laparoscopic surgery in this period have been shown to reduce the incidence of postoperative infections. Moreover, most of the previous systematic reviews and meta-analysis mostly focused upon SSI alone, whereas this analysis also determined the effect of OABs on AL. 

Bacteria have been shown to play a major role in the pathogenesis of anastomotic insufficiency and SSI. Excessive peri-operative contamination of intestinal contents can lead to SSIs, such as in cases of microperforation or subclinical anastomotic leak. Furthermore, bacterial products may cause local inflammation at the anastomosis with intramural abscess formation and anastomotic dehiscence [51]. There is emerging evidence suggesting that certain pathogens, such as *Enterococcus faecalis* and *Pseudomonas aeruginosa*, play a causative role in the development of anastomotic leaks [52,53]. One of the mechanisms underlying the development of AL is probably the capacity of these intestinal bacteria to degrade collagen [52]. Recent work in a rat model demonstrated that virulent strains of *Enterococcus faecalis* can take advantage of a depleted post-operative colonic microbiome and contribute to AL via MMP-9 activation and collagenase expression. *E. faecalis* has been shown to degrade collagen I and activate MMP9, which in turn degrades collagen IV [54]. Both collagen I and IV play an important role in maintenance and repair of the extracellular matrix [55]. A different microbiome in the right-sided colon, with fewer collagenase producing bacteria, may be one of the factors causing less anastomotic leakage in the right-sided colonic surgeries. 

Concerning the oral antibiotic regimes, both selective [17,40] and broad-spectrum OABs have been reported in the included studies (Table 1). SDD is known to target only specific (aerobe, Gram-negative) bacteria while leaving indigenous anaerobic bacteria largely undisturbed [56,57,58]. The disadvantage of broad-spectrum OABs is that they result in a more extensive elimination of bacteria, possibly leading to microbial dysbiosis. With this study we were able to show that SDD combined with MBP was associated with a statistically significant risk reduction in AL compared to broad-spectrum OABs in addition to MBP. SDD appears to be more effective compared to broad-spectrum antibiotics because it covers more specifically the PPMs. This could also be the reason we observed a larger reduction of infectious complications (SSI and AL) in the present meta-analysis compared to other studies, because our meta-analysis included three large studies using SDD [17,41].

The gut microbiota varies widely between individuals [9]. Therefore, it appears plausible that one OAB regime may have variable effects on the diverse microbiome of different patients and, thereby, on prevention of postoperative infectious complications. To address this problem, studies are needed that map the microbiome of the included patients and attempt to correlate this to rates of infectious complications.

The role of MBP on infectious complications is a complex one. Our study could not provide more insight into the effect of MBP because, in all included studies, both patients of the control and intervention groups used MBP. The rationale for its administration is to clean the gut of feces, because the volume hinders the surgeon during the procedure, as it is also hypothesized that it reduces the microbial burden present within the colonic lumen and mucosa, thus preventing direct microbial contamination of the operative site [59]. MBP is often only used in left-sided colon surgery, because the right colon is expected to have less of a stool burden volume compared to the left colon or rectum.

The present study has several limitations. First, there were variations in i.v. antibiotics and OABs, and in the type of MBP used. There were also variations in the dosage, duration of antibiotics used before surgery, and continuation of antibiotics after surgery, thus limiting our ability to comment on the optimal choice. Secondly, the use of enhanced recovery protocols is not always documented, and these are also known to impact patient outcomes. Thirdly, there are limited data on the value of preoperative OABs in the unprepared colon, with one cohort study finding no benefit [29], and a further two studies reporting a reduction in SSI rates [28,60]. Therefore, we were unable to distinguish whether the reduction in SSI and AL is a result of OABs on their own or in combination with MBP.

Despite these limitations, our study has several key strengths. The main strength of this systematic review and meta-analysis is that, to the best of our knowledge, it is the first study evaluating the role of OABs on SSI and AL in a homogeneous group of patients undergoing elective CRC surgery in a contemporary setting. Even with this focused review, the size of our study was still substantial. Furthermore, another strength of this meta-analysis is that it also specifically reports the effects of SDD and broad-spectrum OABs separately on SSI and AL. To ensure that the inclusion of studies using a less rigorous methodology did not exert an undue bias, a predetermined analysis of studies using the Cochrane Collaboration risk of bias tool was used. The risk of bias for the randomized controlled trials included in the meta-analysis was assessed and found to vary. Whereas random sequence generation was of high quality in almost all studies, the high risk of bias in most studies was seen in the blinding of participants, clinicians, or researchers, and the blinding of the outcome assessment. Overall study heterogeneity was low (I^2^ = 0.00–1.03%) for all clinical outcome measures, suggesting that heterogeneity had no effect on the outcome of our meta-analysis. 

## 5. Conclusions

This systematic review and meta-analysis demonstrate that preoperative OAB prophylaxis, in combination with MBP and standard i.v. antibiotic prophylaxis, is associated with a significant reduction in rates of SSI and AL. Furthermore, OABs as SDD seems to be more effective compared to broad-spectrum antibiotics in reducing the risk of SSI and AL after CRC surgery. Emerging research focusing on the microbiome is likely to guide more personalized and specific bowel preparation regimes, which will target reduction of both SSI and anastomotic leakage. 

## Figures and Tables

**Table 1:** Baseline characteristics per study.

Reference and Year of Publication	Study Methodology	Number of Patients	Intervention Group (N)	Control Group (N)	Endpoint SSI	Endpoint AL	Type of Resection	Laparoscopic/Open Surgery	OAB Agent	MBP Agent	Intravenous (i.v) Antibiotics	Comparison Included
Takesue et al., 2000 [35]	RCT	83	38	45	Yes	Yes	Ileocecal resection–5 Right colectomy–14 Left colectomy–3 Transverse colectomy–6 Sigmoidectomy–24 LAR–24 Miles’ APR–7	Open	Kanamycin 500 mg + Metronidazole 500 mg at 2 p.m., 3 p.m. and 11 p.m. when surgery was scheduled on 9 a.m. + control group treatment	Polyethylene glycol at 10 a.m. the day before surgery	Cefmetazole 1 g after induction of anesthesia, administered three times a day for 3 consecutive days	MBP + OAB vs. MBP
Ishida et al., 2001 [36]	RCT	143	72	71	Yes	Yes	Colectomy–76 Anterior resection–47 APR–9 Total proctectomy with J pouch–3 Total pelvic exenteration–4 Other–4	Unknown	Kanamycin 500 mg + Erythromycin 400 mg in 4 daily doses, started 2 days preoperatively + control group treatment	Polyethylene glycol 2 L given the day before surgery	Cefotiam 1 g in 2 daily doses for 48 h	MBP+OAB vs. MBP
Kobayashi et al., 2007 [37]	RCT	484	242	242	Yes	No	Surgical procedure: Colon–241 Rectum–243	Unknown	Kanamycin 1 gr + Erythromicin 400 mg at 14:00, 15:00, and 23:00 + control group treatment	Polyethylene glycol 2 L given the day before surgery	Cefmetazole 1 g after the induction of anesthesia, additional dose if the operation was prolonged beyond 3 h. Again twice daily for 3 consecutive days	MBP+OAB vs. MBP
Roos et al., 2009 [40]	Cohort	162	76	86	Yes	Yes	Hemicolectomy (right sided)–42 Hemicolectomy (left sided)–15 Transversectomy–4 (Subtotal) colectomy–3 Sigmoid resection–38 LAR–43	Combination	Polymyxin B sulphate 100 mg + Tobramycin 80 mg + Amphotericin B 500 mg 4 daily doses, started 2 days preoperatively + control group treatment	Two to 4 L of Klean-Prep^®^ were administered in 24 h and/or a fluid diet was given starting 1 day before surgery. In rectal surgery, also an enema was applied	Cefuroxime 1500 mg + metronidazole 500 mg 3 doses in 24 h	MBP+OAC vs. MBP
Hata et al., 2016 [38]	RCT	579	289	290	No	Yes	Colectomy–376 Anterior resection–183 APR–20	Laparoscopic	Kanamycin 1 g + Metronidazole 750 mg at 13 h and 9 h before the surgery + control group treatment	Sodium picosulphate 75 mg and magnesium citrate 34 g with 180 mL water the day before surgery	Cefmetazole 1 g was administered intravenously 30 min before the skin incision, additional dose was given every 3 h during the surgery	MBP+OAB vs. MBP
Ikeda et al., 2016 [39]	RCT	511	255	256	No	Yes	Colonic surgery–309 Anterior resection–177 APR–25	Laparoscopic	Kanamycin 1000 mg 2 doses + Metronidazole 750 mg, started 1 day preoperative + control group treatment	Magnesium citrate and sodium picosulphate the day before surgery	Cefmetazole 1 g 3 doses in 24 h	MBP+OAB vs. MBP
Abis et al., 2019 [17]	RCT	455	228	227	Yes	Yes	Right hemicolectomy–162 Transverse colectomy–17 Left hemicolectomy–41 Sigmoid resection–124 Low anterior resection–103 Other–8	Combination 98.2% laparo-scopic and 1.8% open in both groups	SDD 3 days prior to surgery until 3 days after surgery or when normal bowel motion occured + control group treatment	Klean-Prep	Cefazoline 1 gr + Metronidazol 500 mg, intravenously, 30 min prior to skin incision	MBP+OAB vs. MBP
Schardey et al., 2020 [41]	RCT	80	40	40	Yes	Yes	(low anterior resection with TME–80	Unknown	Polymyxin B sulphate 100 mg + Tobramycin 80 mg + Vancomycin 125 mg + Amphotericin B 500 mg 4 daily doses, started 1 day preoperatively till day 7 postoperatively.	Klean-Prep	Amphotericin B 500 mg + Lactulose 305 mg	MBP+OAB vs. MBP

**Table 2:** Risk of bias within randomized controlled trials included within the meta-analysis.

Reference	Random Sequence Generation	Allocation Concealment	Blinding of Participant and Personnel	Blinding of Outcome Assessment	Incomplete Outcome Data	Selective Reporting
Takesue et al. [35]	unclear	unclear	unclear	unclear	high	unclear
Ishida et al. [36]	low	high	high	high	low	unclear
Kobayashi et al. [37]	low	unclear	high	high	high	high
Hata et al. [38]	low	low	high	high	low	low
Ikeda et al. [39]	low	low	high	low	low	low
Abis et al. [17]	low	low	high	low	low	low
Schardey et al. [41]	low	low	low	low	high	high
